# Dinaciclib, a Bimodal Agent Effective against Endometrial Cancer

**DOI:** 10.3390/cancers13051135

**Published:** 2021-03-06

**Authors:** David Howard, David James, Kate Murphy, Jezabel Garcia-Parra, Belen Pan-Castillo, Stuart Rex, Annemarie Moul, Eilir Jones, Marc Bilbao-Asensio, Saul Michue-Seijas, Kerryn Lutchman-Singh, Lavinia Margarit, Lewis W. Francis, Paul Rees, Deyarina Gonzalez, R. Steven Conlan

**Affiliations:** 1 Medical School, Swansea University, Singleton Park, Swansea SA2 8PP, UK; david.howard@swansea.ac.uk (D.H.); d.w.james@swansea.ac.uk (D.J.); j.garciaparra@swansea.ac.uk (J.G.-P.); belen.pan.castillo@gmail.com (B.P.-C.); l.francis@swansea.ac.uk (L.W.F.); d.gonzalez@swansea.ac.uk (D.G.); 2 Department of Pathology, Singleton Hospital, Swansea Bay University Health Board, Swansea SA2 8QA, UK; Kate.Murphy@wales.nhs.uk (K.M.); stuart.rex@wales.nhs.uk (S.R.); annemarie.moule@wales.nhs.uk (A.M.); eilir.jones2@wales.nhs.uk (E.J.); 3 Department of Chemistry, College of Science, Swansea University, Singleton Park, Swansea SA2 8PP, UK; 971253@swansea.ac.uk (M.B.-A.); saul.michue-seijas@swansea.ac.uk (S.M.-S.); 4 Department of Gynaecology Oncology, Singleton Hospital, Swansea Bay University Health Board, Swansea SA2 8QA, UK; Kerryn.Lutchman-Singh@wales.nhs.uk; 5 Department of Obstetrics and Gynaecology, Princess of Wales Hospital, Cwm Taf Morgannwg University Health Board, Bridgend CF31 1RQ, UK; Lavinia.Margarit@wales.nhs.uk; 6 College of Engineering, Swansea University, Bay Campus, Swansea SA1 8EN, UK; p.rees@swansea.ac.uk

**Keywords:** endometrial cancer, CDK inhibitor, dinaciclib

## Abstract

**Simple Summary:**

Endometrial cancer (EC) is diagnosed in almost 400,000 women every year globally and is the sixth most common cancer in women. When diagnosed early, treatment can result in a full recovery; however, when at an advanced stage, fewer than 50% of patients survive beyond 5 years and new treatments are needed for these patients. The aim of this investigation was to evaluate cyclin-dependent kinase inhibitors (CDKis) for the treatment of EC using cells isolated directly from patient tumors and cell lines. We compared several CDKis and found one CDKi, dinaciclib, to be particularly toxic to EC cells. Dinaciclib both prevented EC cells from proliferating and blocked transcriptional activity. The drug was equally effective across EC subtypes and combined effectively with cisplatin. This study highlights the potential clinical benefit of dinaciclib for use in the treatment of EC.

**Abstract:**

Endometrial cancer (EC) is the sixth most prevalent female cancer globally and although high rates of success are achieved when diagnosed at an early stage, the 5-year survival rate for cancers diagnosed at Stages II–IV is below 50%. Improving patient outcomes will necessitate the introduction of novel therapies to the clinic. Pan-cyclin-dependent kinase inhibitors (CDKis) have been explored as therapies for a range of cancers due to their ability to simultaneously target multiple key cellular processes, such as cell cycle progression, transcription, and DNA repair. Few studies, however, have reported on their potential for the treatment of EC. Herein, we examined the effects of the pan-CDKi dinaciclib in primary cells isolated directly from tumors and EC cell lines. Dinaciclib was shown to elicit a bimodal action in EC cell lines, disrupting both cell cycle progression and phosphorylation of the RNA polymerase carboxy terminal domain, with a concomitant reduction in Bcl-2 expression. Furthermore, the therapeutic potential of combining dinaciclib and cisplatin was explored, with the drugs demonstrating synergy at specific doses in Type I and Type II EC cell lines. Together, these results highlight the potential of dinaciclib for use as an effective EC therapy.

## 1. Introduction

Endometrial cancer (EC) is the sixth most prevalent cancer in women globally with 382,069 EC diagnoses and 89,929 mortalities related to the disease in 2018 [1]. The disease has traditionally been classified into two subtypes (Types I and II), reflecting histopathological and molecular differences that inform prognosis and clinical decision making. Type I disease arises from endometrial hyperplasia caused by excess estrogen exposure, either due to physiological factors such as obesity, early menarche/late menopause, and nulliparity, or from exogenous sources, such as hormone replacement therapy, or tamoxifen therapy, which display estrogenic activity in the uterus [2]. Type I tumors have an endometrioid histology and are typically estrogen- and progesterone-receptor-positive [3]. Approximately, 90% of EC diagnoses are for Type I disease and these cancers typically present at an early stage and have a positive prognosis with a 5-year survival of 75% [2]. The remaining 10% of EC diagnoses are designated Type II and comprise serous, clear cell, carcinosarcoma, and squamous tumor histologies. Serous tumors arise from a serous endometrial intraepithelial carcinoma (SEIC) precursor; however, the origins of the remaining histological subtypes are not known [4]. Unlike Type I disease, Type II EC is not associated with estrogen exposure [3,4]. Type II ECs are aggressive, often diagnosed at a late stage, and collectively carry a 5-year survival rate of just 55% [2].

Treatment for EC involves hysterectomy with bilateral salpingo-oophorectomy for Stage I disease, with more extensive surgical resection necessary at later cancer stages [5]. Adjuvant therapy in the form of radiotherapy, chemotherapy (typically taxane or platinum-based), or a combination is used for Stage III and IV disease, and for Stage I and II where there is a risk of recurrence [6]. In cases where surgery is not feasible, radiotherapy, sometimes in combination with chemotherapy, is performed as a neoadjuvant therapy, with surgical resection following where possible [7]. Targeted therapies including PI3K/AKT/mTOR pathway inhibitors, HER2 (human epidermal growth factor receptor 2), EGFR (epidermal growth factor receptor) inhibitors and aromatase inhibitors have been trialed for advanced EC; however, the majority of these therapies have demonstrated little efficacy and few have progressed beyond Phase II trials [8]. Thus, it is clear that improving outcomes for patients afflicted with both Type I and Type II EC requires novel therapeutic approaches.

Cyclin dependent kinases (CDKs) are a family of kinases, which, in complex with an activating member of the cyclin protein family, play roles in cell cycle progression, transcription, and signal transduction and are therefore active upstream of myriad cellular processes [9]. Twenty-one CDKs have been identified in humans to date, amongst which CDKs 1, 2, 4, and 6 are involved in cell cycle progression and CDKs 7, 8, 9, 12, 13, and 19 in transcriptional regulation [10]. The dependency of tumors on transcription for growth and survival and efficient cell cycle progression for rapid proliferation make these CDKs attractive therapeutic targets, and CDK inhibitors (CDKis) are currently under evaluation in numerous cancers [10]. CDK inhibitors (CDKis) targeting cell cycle CDKs have been shown to block tumor proliferation through cell cycle arrest [11,12], while the anti-tumor effects for CDKis targeting transcriptional CDKs have been reported to arise from reduced expression of oncogenes [13], DNA repair [14], and anti-apoptotic proteins [15,16].

To date, the success of CDKis in the clinic has been mixed. Third-generation CDKis, which are selective CDK4/6 inhibitors including abemaciclib, palbociclib, and ribociclib, are in clinical use for hormone receptor-positive, HER2-negative advanced breast cancer [17]. In contrast, pan-CDKis, so described because they target multiple CDKs, have yet to receive clinical approval, and tolerability issues have led to multiple failing in clinical trials [18]. Trials for pan-CDKis, however are still ongoing [19,20] and their ability to simultaneously target CDKs involved in cell cycle and transcription suggest they might be more versatile and efficacious than the selective CDK4/6 inhibitors. The genetic landscape of EC suggests it may be particularly susceptible to pan-CDKis, which target both cell cycle and transcriptional CDKs. Approximately 85% of Type II EC tumors harbor altered expression of cell-cycle-related genes [21], including amplification of CCNE1 (48% of cases) [22], the gene encoding cyclin E, which, in complex with CDK2, drives G1/S progression. The role of estrogen in the tumorigenesis of Type I EC is driven by increased cyclin D expression driving increased CDK 4/6 activity, thereby resulting in increased cell proliferation [23,24,25]. Mutation-driven dysregulation of the MAPK/ERK and WNT1 pathways are common features of Type I EC tumors, which also promote proliferation through CDK 4/6–cyclin D activity [26]. Overexpression of cyclin D has been observed in both Types I and II EC, where it is associated with poor prognosis [27]. Furthermore, overexpression of the anti-apoptotic genes BCL2 [28], and BCL2L2 [29] has been reported in EC, where they may promote tumor cell survival. Beyond the theoretical benefits of suppressing the expression of EC oncogenes (such as PI3K, KRAS, CTNNB1, ERBB2, and MYC) [30], CDKis may also offer clinical benefits through reducing the expression of anti-apoptotic genes in these tumors.

Here, we investigated the utility of the CDK inhibitors DRB (6-dichloro-1-beta-D-ribofuranosylbenzimidazole), flavopiridol, and dinaciclib for the treatment of EC. DRB is a specific inhibitor of the transcriptional kinase CDK9. Flavopiridol and dinaciclib target both transcriptional and cell cycle CDKs, with flavopiridol inhibiting CDKs 1, 2, 4, 6, and 9 and dinaciclib inhibiting CDKs 1, 2, 5, 9, and 12 [31,32]. Our results demonstrated that EC cells were most sensitive to dinaciclib and that dinaciclib was effective against primary tumor cells isolated from both Type I and II EC patients. Dinaciclib displayed a bimodal molecular effect, both blocking cell cycle progression and reducing phosphorylation of RNA Polymerase II (Pol II) *C*-terminal domain (CTD), with a concomitant reduction in the expression of anti-apoptotic genes. Furthermore, dinaciclib demonstrated synergy with cisplatin in Type I and Type II EC cell lines.

## 2. Materials and Methods

### 2.1. Cell Culture

Ishikawa and HEC50 cells were purchased from the European Collection of Cell Cultures (ECCAC, Porton Down, UK), and HEC-1A and HEC-1B cells were purchased from the American Type Culture Collection (ATCC, Manassas, VA, USA). Cell lines were cultured in plastic culture flasks (Porvair Sciences, Wrexham, UK) using Dulbecco’s Modified Eagle Medium: Nutrient Mixture F-12 (DMEM-F12) supplemented with 10% fetal bovine serum (FBS), 1 mM sodium bicarbonate and 1% penicillin–streptomycin (all from Gibco by ThermoFisher Scientific, Waltham, MA, USA). Cells were maintained at 37 °C in a 5% CO_2_ humidified incubator.

### 2.2. Isolation and Expansion of Patient Tumor-Derived Cells

Endometrial tumor cells were isolated and propagated from tumor biopsies obtained from consenting patients under ethical approval provided from the Local Ethics Committee (LREC), Wales 6, ref 07/WMW02/50. Tumors were formalin-fixed and paraffin-embedded, and tissue sections were analyzed by immunohistochemical detection, as previously described [33] using anti-p53 (M7001) (Dako, Jena, Germany) and anti-ER (Roche 790-4324) (Ventana Systems, Oro Valley, AZ, USA) antibodies. Scoring of immunohistochemical (IHC) staining was performed as previously described [33]. Tissue samples were delivered in centrifuge tubes in DMEM-F12 medium (Gibco by ThermoFisher). On the day of receipt, tissue samples were disrupted in a petri dish by thorough chopping with a scalpel. DMEM-F12 supplemented with 10% fetal bovine serum (FBS), 1.5 mM glutamine, 1 mM sodium bicarbonate, 1 mM sodium pyruvate, and 1% penicillin–streptomycin (all from Gibco, by Thermo Fisher Scientific) was added to the tissue. To reclaim any detached cells present in the medium in which the biopsy was delivered, this fraction was centrifuged at 200× *g* to form a cell pellet. The supernatant was discarded, and the cell pellet was resuspended in 1 mL of the medium and added to the dish containing tissue. The tissue/cell mixture was maintained at 37 °C in a 5% CO_2_ humidified incubator and monitored daily. Approximately 4 days following tissue processing, the petri dish was washed to remove non-adherent cells and tissue detritus, and fresh medium was added. Cells were then expanded before being used in drug treatment experiments.

### 2.3. Cell Viability

Dinaciclib (Selleckchem, Houston, TX, USA), flavopiridol (Sigma-Aldrich, St Louis, MO, USA), and DRB (Sigma-Aldrich) were dissolved in DMSO (dimethyl sulfoxide) to make 10 mM stock solutions, and cisplatin (Sigma-Aldrich) was dissolved in saline to make a 3 mM stock solution. Cells were seeded in white-walled 96-well plates (Porvair Sciences, Wrexham, UK) at densities of 500 cells/well for cell lines and 750 cells/well for primary cells. Twenty-four hours following seeding, the medium was removed and replaced with a medium containing the drug or the vehicle control. The treatment medium was additionally supplemented with RealTime-Glo MT Cell Viability Assay (Promega, Madison, WI, USA) reagents at manufacturer-recommended concentrations (1:1000). Treated samples were kept in a cell culture incubator and luminescence per well was measured every 24 h in a microplate photometer at 37 °C.

### 2.4. Trypan Blue Live/Dead Cell Assay

Cells were seeded in 6-well tissue culture plates at densities of 300,000, 200,000 and 100,000 cells per well for 24-, 48-, and 72-h treatments. Cells were treated with dinaciclib at 10 and 40 nM doses or with DMSO (Sigma-Aldrich) as a vehicle control. Treatments were performed in individual culture plates for 24, 48, and 72 h, and cells were counted at the end of treatments. To count cells, the medium was first removed from all wells and added to a centrifuge tube, in order to recover any non-adherent (presumably dead) cells. Adherent cells were suspended by incubation for 5 min with 0.5 mL 0.25% trypsin (in 1 mM ethylenediaminetetraacetic acid (EDTA) (pH 8) in Hank’s Balanced Salt Solution (HBSS)) (Gibco by ThermoFisher Scientific) in a cell culture incubator. Wells were examined under a microscope to ensure all cells were detached before combining the resulting cell suspension to the centrifuge tube containing the corresponding initial treatment medium. The cell suspensions were pelleted by centrifugation at 200× *g* for 5 min, the medium supernatant was carefully removed by pipette, and the remaining pellet was resuspended in 1 mL of cell medium. To identify dead cells, 20 μL of the cell suspension from each sample was mixed with an equal volume of Trypan Blue solution (0.4%) (Sigma-Aldrich) and incubated for 1 min ahead of cell quantification using a TC20 Automated Cell Counter (Bio-Rad, Hercules, CA, USA).

### 2.5. RealTime-Glo Annexin V Apoptosis and Necrosis Assay

The RealTime-Glo Annexin V Apoptosis and Necrosis Assay (Promega, Madison, WI, USA) was used to quantify early- and late-stage apoptosis and was performed as an endpoint assay following the manufacturer’s instructions. Ishikawa and HEC cells were seeded with a density of 2000 cells/well in white-walled 96-well plates (Porvair Sciences, Wrexham, UK) in 100 μL medium and incubated at 37 °C in 5% CO_2_ humidified air for 24 h. Cells were then treated with DMSO or dinaciclib at 10, 40, and 80 nM concentrations for 12 and 24 h durations. The response of Ishikawa and HEC-1A cells to staurosporine (2 µM) (Sigma-Aldrich) was measured following 24 h of treatment. Luminescence and fluorescence (530 nm emission filter) was measured using a microplate photometer. Wells were then washed twice in PBS, and a cell viability assay was performed as described above in order to quantify viability per well relative to the control wells.

### 2.6. Cell Cycle Analysis

Cells were seeded in 6-well plates at densities of 200,000 cells/well and were treated for 24 h following with a vehicle control or dinaciclib at 10 and 40 nM concentrations. Cells were then washed in PBS and fixed by incubation with a 4% formaldehyde solution (Sigma-Aldrich). Hoechst 33342 (ThermoFisher Scientific) in PBS at 5 μg/mL was added to the wells and the plates incubated for 4 h in the dark. Cells were washed twice more in PBS and then 3 mL of PBS was added to each well ahead of imaging. Imaging was performed using the In Cell 2000 (GE Healthcare, Chicago, IL, USA) high-throughput imaging system with 120 images taken per well using a 20× objective and 450/65 nm emission filter. Images were processed using Cell Profiler 3.0.0 (Broad Institute, Cambridge, MA, USA) [34]. Nuclei were segmented by object diameter, and Otsu thresholding and the integrated object intensity for each nucleus were recorded. A histogram of integrated object intensity vs. the number of objects was generated for each sample in MATLAB (MathWorks, Natick, MA, USA). G1, G2/M, and S phase peaks were fitted to the nuclear integrated intensity histograms using the Watson Pragmatic algorithm [35], and integration of the peaks yielded the percentages of nuclei per cell cycle phase. Three experimental replicates were performed per sample.

### 2.7. Immunoblotting

Ishikawa and HEC-1A cells were grown in T75 flasks and treated with dinaciclib (10 or 40 nM) or the vehicle control for periods of 4 and 20 h. Cells were then extracted in a RIPA (Radio-Immunoprecipitation Assay) buffer (Sigma-Aldrich) supplemented to 1% *v*/*v* each of Proteinase Inhibitor Cocktail 1, and Phosphatase Inhibitor Cocktails 2 and 3 (all from Sigma-Aldrich) and transferred into 1.5 mL microcentrifuge tubes. Samples were incubated on ice for 30 min with intermittent agitation (vortexing) to lyse cells. Samples were centrifuged at 20,000× *g* for 10 min at 4 °C to pellet cell debris, and the supernatant containing the protein fraction was retained. Protein samples were quantified using the DC Protein Assay (Bio-Rad, Hercules, CA, USA). To prepare protein samples for separation, 30 µg of protein was heated at 95 °C for 5 min in a Laemmli sample buffer containing 5% *v*/*v* β-mercaptoethanol (Sigma-Aldrich). Protein samples were then separated through sodium dodecyl polyacrylamide gel electrophoresis (SDS-PAGE) using Mini-PROTEAN TGX Precast 4–20% gels (Bio-Rad) and then transferred onto polyvinylidene difluoride (PVDF) membranes using the Trans-Blot Turbo transfer system (Bio-Rad). Membranes were blocked for 1 h at RT (room temperature) in 5% bovine serum albumin (BSA) (PAN Biotech, Aidenbach, Germany) in Tris-buffered saline (TBS) with 0.1% Tween-20 (Sigma-Aldrich) and then probed overnight with anti-Pol II, Ab817 (Abcam, Cambridge, UK), anti-Pol II pSer2, 61083 (Active Motif, Carlsbad, CA, USA), anti-MPM2, 05-368 (Merck Millipore, Burlington, MA, USA), and anti-GAPDH, sc25778 (Santa Cruz Biotechnologies, Dallas, TX, USA), antibodies. In some cases, the membranes were cut according to molecular weight and antibodies were applied separately to avoid stripping and reprobing (original membranes are shown as supplementary data). Following washes (4 × 5 min) in TBS/Tween-20, membranes were incubated with appropriate horseradish peroxidase- (HRP)-conjugated secondary antibodies (NA931V and NA934, both from GE Healthcare, Chicago, IL, USA) and sc2032 (Santa Cruz Biotechnologies) for the detection of mouse, rabbit, and rat antibodies, respectively, for 1 h at RT. Protein bands were detected and imaged using Clarity Western Enhanced Chemi Luminescence (ECL) substrate and a ChemiDoc Imager (both supplied by Bio-Rad). Densitometry was performed with ImageLab software (Bio-Rad).

### 2.8. qPCR

RNA was extracted from control and dinaciclib-treated samples using the RNeasy Mini Kit (Qiagen, Hilden, Germany) according to the manufacturer’s protocol, from which cDNA was generated using the High Capacity cDNA Reverse Transcriptase Kit (Applied Biosystems by Thermo Fisher Scientific). Reverse transcription reactions were performed using the T100 Thermal Cycler (Bio-Rad). Samples were analyzed by qPCR in triplicate using the iTaq Universal SYBR Green Supermix (Bio-Rad), run on the CFX96 Real-Time PCR Detection System (Bio-Rad) using previously described primers for RPS18 (forward: ATTGCCGACAGGATGCAGAA, reverse: GCTGATCCACATCTGCTGGAA), Mcl-1 (forward: TGATCCATGTTTTCAGCGAC, reverse: AATGGTTCGATGCAGCTTTC), Bcl-2 (forward: GATGTGATGCCTCTGCGAAG, reverse: GATGTCTCTGGAATCT), and survivin (forward: ACCGCATCTCTACATTCAAG, reverse: CAAGTCTGGCTCGTTCTC) [36,37,38,39]. Calibration curves using serial dilutions of cDNA were plotted, and gene expression was quantified by plotting threshold cycle values. Values obtained from the reference gene RPS18 were used to normalize expression across samples. Relative expression was expressed as the mean fold induction ± standard deviation.

### 2.9. Statistical Analysis

The data distribution was assessed for normality using the Ryan–Joiner and Kolmogorov–Smirnov tests using Minitab v13 (Minitab, State College, PA, USA). Non-normally distributed data were analyzed with the non-parametric Kruskal–Wallis test, followed by a Mann–Whitney *U*-test applied post hoc to determine statistical significance. Normally distributed data were analyzed either by the *t*-test or by analysis of variance (ANOVA) followed by Dunnett’s test using Prism v6 (Graphpad, San Diego, CA, USA), where *p* < 0.05 was considered significant.

## 3. Results

### 3.1. CDKis Inhibit Growth in EC Cell Lines and Primary Cells

**Figure 1:**
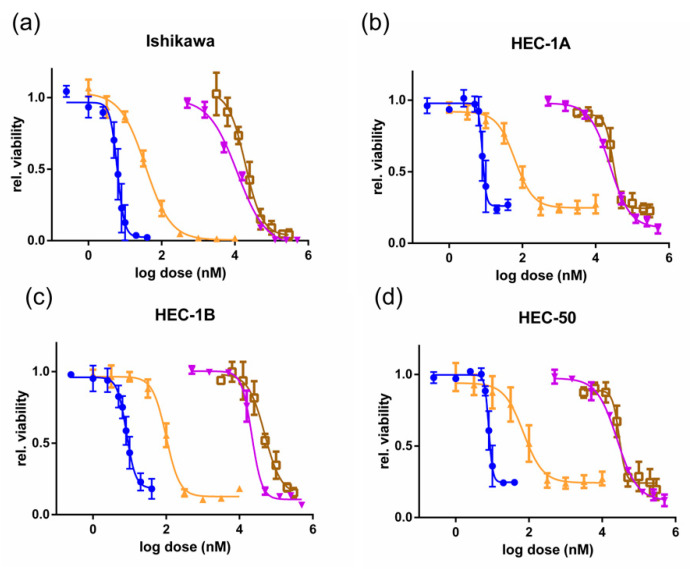
Type I and II endometrial cancer (EC) cell response to dinaciclib, flavopiridol, 6-dichloro-1-beta-D-ribofuranosylbenzimidazole (DRB), and cisplatin exposure. Dose–response experiments are shown following 72 h of treatment with dinaciclib (blue), flavopiridol (orange), DRB (brown), and cisplatin (purple) in the Type I EC cell line (**a**) Ishikawa and the Type II EC cell lines (**b**) HEC-1A, (**c**) HEC-1B, and (**d**) HEC-50. Viability was measured using a RealTime-Glo MT cell viability assay and is normalized to vehicle controls.

Platinum-based drugs are a mainstay of EC therapies [6]. Cisplatin was therefore used as a benchmark to compare the efficacies of CDK-inhibiting compounds. Dose–response experiments measuring cell viability (Figure 1) were performed in the Type I EC cell line Ishikawa, and the Type II EC cell lines HEC-1A, HEC-1B, and HEC-50. The shift in the curves between the treatments along with the drug IC50s (half maximal inhibitory concentrations) (Table S1) highlight the relative potencies of these agents. Ishikawa Type I EC cells were the most sensitive to cisplatin, with an IC50 of 11 µM, whereas in Type II EC cells, the IC50 was 28 µM (HEC-1A), 24 µM (HEC-1B), and 29 µM (HEC-50). The CDK9 inhibitor DRB inhibited proliferation at similar concentrations to cisplatin with IC50s of 17, 42, 39 and 41 µM in Ishikawa, HEC-1A, HEC-1B, and HEC-50 cells, respectively. The pan-CDK inhibitors flavopiridol and dinaciclib proved much more potent than cisplatin, with IC50s in the nM range. Flavopiridol had an IC50 of 39 nM in Ishikawa cells, and in HEC-1A, HEC-1B, and HEC-50 cells, the IC50s were two- to three-fold greater. Both Type I and Type II EC cell lines were highly sensitive to dinaciclib, with IC50s ranging from 6 to 9 nM across all four cell lines. This contrasts significantly with the much higher IC50s for the current EC therapies carboplatin and doxorubicin (Table S1).

**Figure 2:**
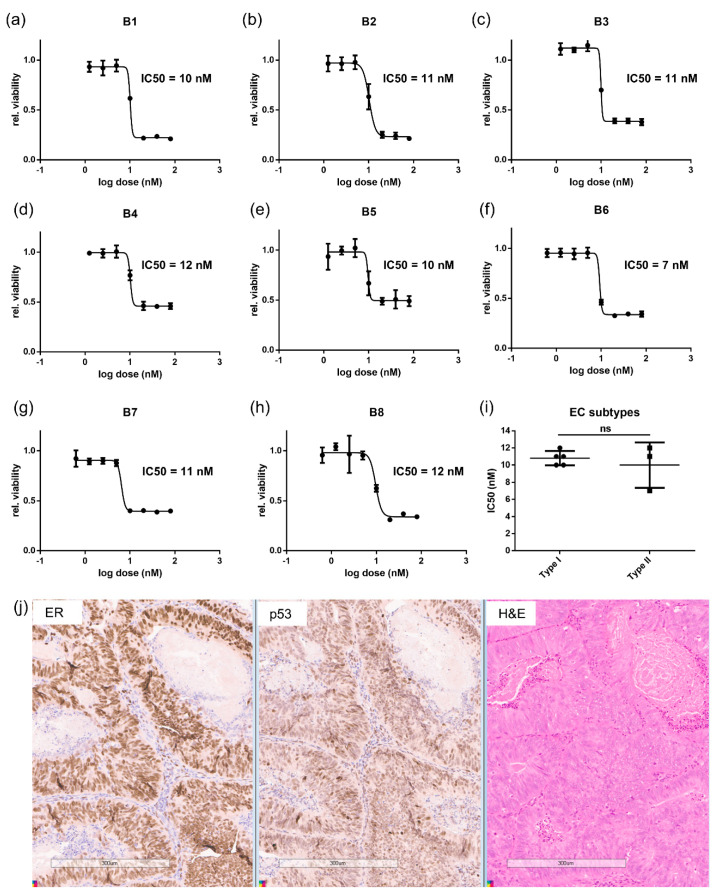
Dinaciclib is effective at inhibiting growth of primary endometrial tumor cells isolated from EC patients. Dose–response following 72 h of treatments with dinaciclib in tumor cells isolated from eight EC tumors, labeled in the graphs as B1 to B8 (**a**–**h**). Viability was measured using a RealTime-Glo MT Cell Viability Assay and normalized to vehicle controls. (**i**) A comparison of the IC50s of tumor cells derived from Type I (B1, B2, B5, B7, B8) and II (B3, B4, B6) tumors. (**j**) Immunohistochemical (IHC) panel showing, from left to right, ER, p53, and H and E (hematoxylin and eosin), staining of EC biopsy tissue, Scale Bar is 300 μm. Statistical significance was calculated using the unpaired *t*-test. ns = not significant.

Based on the high sensitivities of the EC cell lines to dinaciclib, we evaluated its efficacy on primary EC Type I and II tumor cells to determine the clinical relevance of the response. Biopsies from eight patient tumors were classed as either Type I or II based on histology and grade. Immunohistochemical (IHC) analysis of estrogen receptor alpha (ERα) and p53 of the biopsy samples was determined (Figure 2j and Table S2). Cells isolated from these biopsies were treated with dinaciclib in dose–response experiments (Figure 2a–h). All primary cells proved highly sensitive to dinaciclib, with IC50s of 7–12 nM. Moreover, there was no significant difference in sensitivity to dinaciclib between Type I and II primary cells (Figure 2i), further demonstrating the consistent sensitivity of EC cells to dinaciclib.

### 3.2. Dinaciclib Is Cytotoxic to Ishikawa Cells and Blocks the Proliferation of HEC-1A Cells

**Figure 3:**
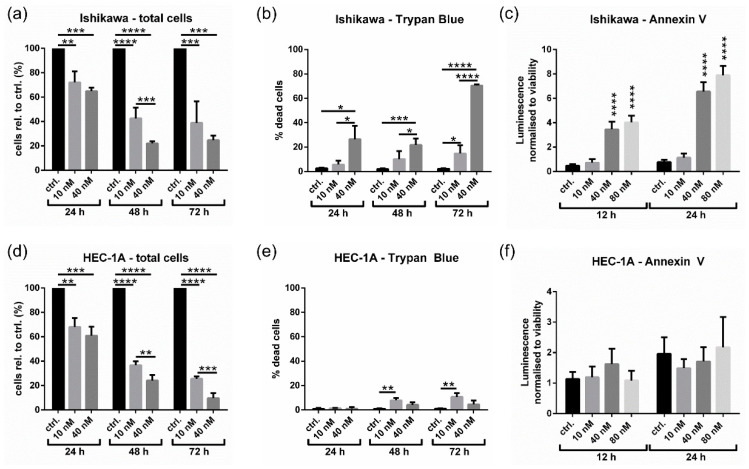
Dinaciclib shows potent anti-proliferative activity in Ishikawa and HEC-1A cells, and is cytotoxic in Ishikawa cells. Total cell numbers were counted using an automated cell counter in (**a**) Ishikawa and (**d**) HEC-1A samples following treatment with DMSO, or 10 or 40 nM dinaciclib for 24, 48, or 72 h. Trypan Blue was used to identify dead cells within total cell counts as shown in (**b**) and (**e**) for Ishikawa and HEC-1A cells, respectively. Cell numbers are expressed as a percentage of control values. Apoptosis in (**c**) Ishikawa and (**f**) HEC-1A samples was measured using the RealTime-Glo Annexin V Apoptosis and Necrosis Assay following 12 and 24 h of treatment with DMSO, or 10, 40, or 80 nM of dinaciclib. Statistical significance was calculated by ANOVA followed by Dunnett’s test. * *p* value < 0.05, ** *p* value < 0.01, *** *p* value < 0.001, **** *p* value < 0.0001.

To determine whether the reduction in cell viability following dinaciclib treatment was anti-proliferative or cytotoxic, a Trypan Blue dead cell assay was performed to detect dead cells within treated samples. Treatment with 40 nM dinaciclib for 72 h reduced cell numbers to 25% of control levels for Ishikawa (Figure 3a) and 10% for HEC-1A (Figure 3d). For Ishikawa cells (Figure 3b), 40 nM dinaciclib caused significant levels of cell death, which reached 70% after 72 h, whereas at 10 nM, only 15% of cells were dead after 72 h. These results indicate that the effect of dinaciclib in Ishikawa cells is largely anti-proliferative at low nM doses, but it becomes cytotoxic at higher concentrations. In HEC-1A cells, the effect of dinaciclib was predominantly anti-proliferative, with only a small increase in cell death observed after 72 h (Figure 3e).

To further assess whether dinaciclib caused apoptosis in Ishikawa and HEC-1A cells, annexin V levels were determined (Figure 3c,f). Dinaciclib induced apoptosis in Ishikawa cells following treatment at 40 nM, increasing levels of apoptosis eight-fold, and this increased up to 10-fold following exposure to 80 nM dinaciclib. Additionally, levels of late-stage apoptosis were found to be four- to six-fold greater in Ishikawa cells following treatment with 40 and 80 nM dinaciclib (Figure S1b). Consistent with annexin V measurements, no apoptosis was observed in HEC-1A cells (Figure S1d), confirming the differences in response between Type I and II EC cell lines. This was further substantiated by gross phenotypic observations, where treated Ishikawa cells displayed a rounded morphology indicative of apoptosis cells, while HEC-1A cells displayed no obvious morphological changes (Figure S1a,c).

HEC-1A cells are resistant to cisplatin-induced apoptosis (Figure 1) [40]; furthermore, HEC-1A cells, unlike Ishikawa cells, do not undergo apoptosis following exposure to the commonly used apoptotic agent staurosporine (Figure S3). Nonetheless, HEC-1A cells were equally sensitive to the growth-inhibitory effects of dinaciclib (Figure 1 and Figure 3). Together, these data suggest that cisplatin and staurosporine resistance do not correlate with a decreased sensitivity to dinaciclib.

### 3.3. Dinaciclib Induces Cell Cycle Arrest

**Figure 4:**
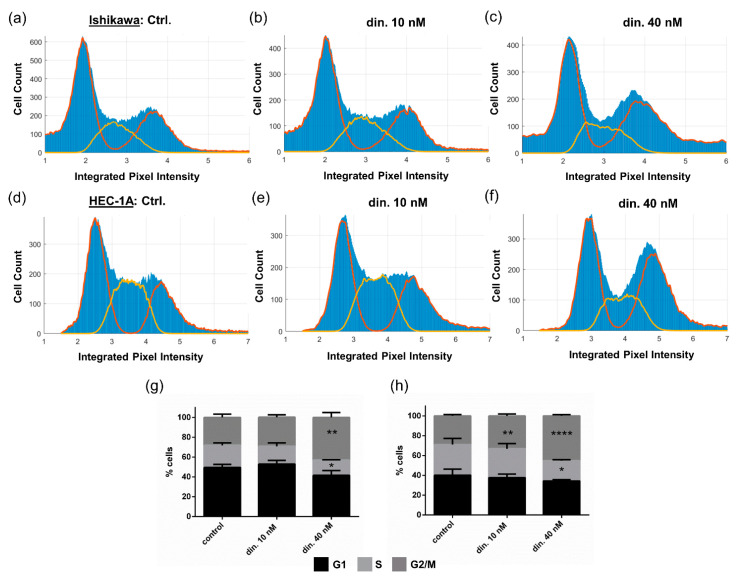
Dinaciclib inhibits cell cycle progression in Ishikawa and HEC-1A cell lines. Cells were treated with either DMSO, or 10 or 40 nM dinaciclib for 24 h, and the nuclei were stained. Nuclear segmentation and DNA content quantification per cell was performed using Cell Profiler. Histograms show (**a**) Ishikawa + control (**b**) Ishikawa + 10 nM dinaciclib, and (**c**) Ishikawa + 40 nM dinaciclib samples, and (**d**) HEC-1A + control, (**e**) HEC-1A + 10 nM dinaciclib and (**f**) HEC-1A + 40 nM dinaciclib samples. The Watson Pragmatic test was used to define G1 and G2/M peaks (red lines on the histograms) and S-phase peaks (yellow lines). The relative percentages of cells in each cell cycle phase (peak integrals) are shown for (**g**) Ishikawa and (**h**) HEC-1A cells. Statistical significance was calculated by ANOVA followed by Dunnett’s test. * *p* value < 0.05, ** *p* value < 0.01, **** *p* value < 0.0001.

In order to establish whether the anti-proliferative effects of dinaciclib were due to cell cycle arrest, cell cycle stage was determined at a cell population level (Figure 4). Treatment with 40 nM dinaciclib caused an accumulation of cells in the G2/M phase in both cell lines, and a small increase in the number of cells in the G2/M phase was seen in HEC-1A samples treated with 10 nM dinaciclib, confirming that arrest was occurring as a result of treatment. We next investigated whether dinaciclib-induced cell cycle arrest occurred within the G2 or M phase by evaluating levels of mitotic protein using the MPM2 (mitotic protein monoclonal-2) antibody, which detects a phospho-epitope present at high levels during mitosis [41]. No general changes in MPM2 levels were observed following dinaciclib treatment indicating that the majority of cells were not in the M phase; however, there was a reduction in the levels of a specific 255 kDa phospho-protein (Figure S2). This large phospho-protein is typically observed on MPM2 immunoblots, and was previously identified as the Ser2 phosphorylated form of RNA polymerase II (pSer2 Pol II) [41]. Such a reduction in pSer2 Pol II would be consistent with dinaciclib targeting CDK9, which, together with Cyclin T, forms the P-TEFb (positive transcription elongation factor) protein complex responsible for Pol II CTD Ser2 phosphorylation during transcription elongation.

### 3.4. Dinaciclib Inhibits Phosphorylation of Pol II CTD at Ser2 and Reduces Expression of Anti-Apoptotic Genes

**Figure 5:**
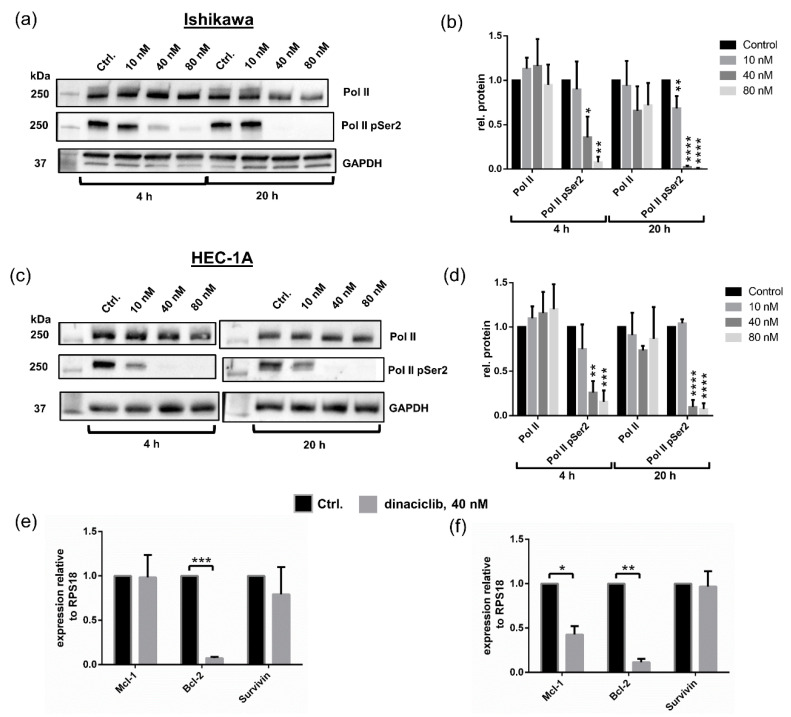
Dinaciclib inhibits Polymerase II (Pol II) *C*-terminal domain (CTD) Ser2 phosphorylation and concomitantly reduces in Bcl-2 expression. (**a**) Immunoblots of Pol II and Pol II pSer2 in Ishikawa cells following 4 h and 20 h of treatment with dinaciclib and the vehicle control and (**b**) the corresponding densitometry data (normalised to Glyceraldehyde-3-phosphate dehydrogenase (GAPDH)). (**c**) Immunoblots of Pol II and Pol II pSer2 in HEC-1A cells following dinaciclib treatment and (**d**) the corresponding densitometry data. qPCR data from (**e**) Ishikawa and (**f**) HEC-1A samples showing Mcl-1, Bcl-2, and survivin mRNA levels following 40 nM dinaciclib treatment for 24 h. Transcript levels are normalised to the reference gene RPS18 and expressed relative to control samples. Statistical significance was calculated by ANOVA followed by Dunnett’s test. * *p* value < 0.05, ** *p* value < 0.01, *** *p* value < 0.001, **** *p* value < 0.0001.

To confirm the effects of dinaciclib on P-TEFb activity, the levels of Pol II and pSer2 Pol II were determined. Pol II pSer2 levels were almost completely eliminated in Ishikawa and HEC-1A samples treated with 40 and 80 nM dinaciclib following 4 h and 20 h of treatments, with 10 nM dinaciclib leading to a significant reduction in Pol II pSer2 levels in HEC-1A cells after 20 h (Figure 5a–d). Next, to investigate the effects of transcription repression, the levels of expression of the anti-apoptotic genes Bcl-2 and Mcl-1 and the survivin gene, a member of the inhibitors of apoptosis (IAP) family, were assessed. Levels of survivin mRNA were unaffected by dinaciclib treatment in both cell lines, while dinaciclib significantly reduced the expression of Bcl-2 to background levels of 6% and 10% in Ishikawa and HEC-1A samples, respectively (Figure 5e,f). Studies in other tumor types have reported that dinaciclib reduces Mcl-1 expression, causing Mcl-1-dependent apoptosis [13,42,43]. Our experiments show that while dinaciclib reduced Mcl-1 expression in HEC-1A cells, it had no effect on Mcl-1 levels in Ishikawa. These results suggest that dinaciclib induces apoptosis in Ishikawa in a Mcl-1-independent manner.

### 3.5. Dinaciclib Sensitizes EC Cell Lines to Cisplatin Treatment

**Figure 6:**
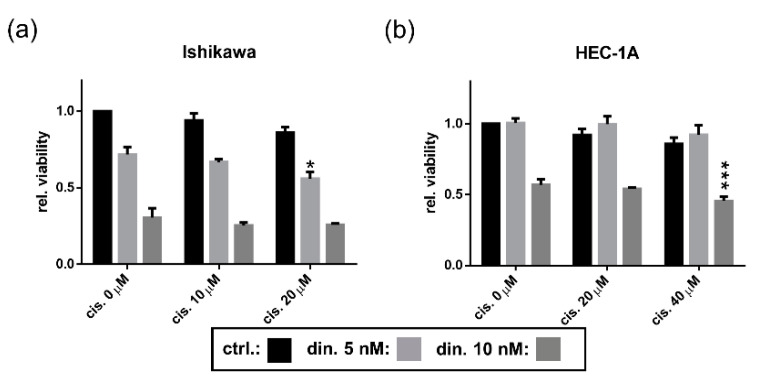
Dinaciclib synergizes with cisplatin in EC cell lines. EC cell lines were treated either individually or in combination with dinaciclib at 5 and 10 nM concentrations and with concentrations of cisplatin reflective of their respective sensitivities (10 and 20 µM for Ishikawa and 20 and 40 µM for HEC-1A). Viability was measured and normalized to vehicle controls for (**a**) Ishikawa and (**b**) HEC-1A cells following 48 h of treatment. A combined treatment was considered synergistic where its viability was significantly lower than the viabilities of samples treated with the corresponding doses of each drug individually. Statistical significance was calculated by ANOVA followed by Dunnett’s test. * *p* value < 0.05, *** *p* value < 0.001.

Dinaciclib has been shown to sensitize cells to cisplatin treatment [44,45,46]. We therefore evaluated the effect of combining cisplatin with dinaciclib on Ishikawa and HEC-1A cells (Figure 6). Whilst it was very evident that the EC cell lines were far more sensitive to dinaciclib than to cisplatin, the drug combination did result in a small decrease in cell viability compared with individual treatments. In Ishikawa cells, a combination of 5 nM dinaciclib and 20 µM cisplatin had a small yet significant effect, with cell viability reduced to 56% compared with only 72% and 86% following individual treatments with dinaciclib and cisplatin, respectively. In HEC-1A, an additive effect was observed at higher treatment levels, where the combination of 10 nM dinaciclib and 40 µM cisplatin resulted in viability being reduced to 46% compared with 57% for dinaciclib-only and 86% for cisplatin-only samples.

## 4. Discussion

The treatment of EC continues to involve radical surgical interventions and adjuvant therapy (radiotherapy or chemotherapy) alone or in combination [6], or, in some cases, with neoadjuvant therapy where surgery is not optimal [7]. The chemotherapies of choice are still taxane or platinum-based drugs due to the relative failure of targeted therapies including PI3K/AKT/mTOR HER2, EGFR, and aromatase inhibitors, with few progressing beyond Phase II trials [8]. The development and application of new treatments for EC therefore remains imperative, particularly as mortality rates are not decreasing for this cancer, in part due to the levels of recurrence in Type I disease and the sparsity of treatment options for Type II disease. The development and utilization of CDK inhibitors has received much attention, particularly for cancer therapeutics, due to the ability of these agents to block cell cycle and therefore cancer cell proliferation [9]. Less prominent has been the exploitation of CDK inhibitors in blocking transcriptional programs, despite the reliance of tumor development on oncogene and gene cluster expression in determining growth and survival.

Here, we explored the bimodal effects of CDK inhibitors in targeting transcription and the cell cycle to evaluate their potential for EC treatment. The anti-proliferative effects of DRB, an inhibitor of CDK9, was compared with those of flavopiridol and dinaciclib, pan-CDK inhibitors targeting both transcription and the cell cycle. In cell lines derived from Type I and Type II EC tumors, all three drugs proved effective in decreasing cell viability; however, the efficacy of dinaciclib was significantly greater than either flavopiridol or DRB, functioning at a sub-10 nM IC50. Dinaciclib was equally effective in significantly decreasing cell viability in both the Type I (Ishikawa) and Type II EC cell models evaluated (HEC-1A, HEC-1B, and HEC-50). Furthermore, the relative resistance of HEC-1A cells to cisplatin did not correlate with any decrease in sensitivity to dinaciclib.

Very importantly, we found that when dinaciclib was evaluated against a small panel of primary tumor-derived cancer cells, the efficacy was essentially the same as for the cell lines, with the IC50 remaining at approximately 10 nM in both Type I and Type II disease. This finding led us to pursue the detailed mechanism of action in the EC cell models, and revealed that high doses (40–80 nM) of dinaciclib induced apoptosis in Type I Ishikawa cells, whilst at a lower 10 nM concentration, its effect was predominantly anti-proliferative. Similarly, in Type II HEC-1A, an anti-proliferative effect was the prevalent mechanism of action. Interestingly, despite differences in cell fate, an identical concentration of dinaciclib was required to reduce viability in the two cell lines. The low-nM effective concentrations of dinaciclib in EC cell lines shown here are far below the peak plasma concentrations recorded in clinical trials with the drug, which, at the tolerated dose of 14 mg/m^2^, ranged between 1 and 2.5 µM [47,48].

The anti-proliferative effects of dinaciclib suggested a cell cycle blockade was occurring. Indeed, as a CDK1 and 2 inhibitor, it was expected that dinaciclib would induce cell cycle arrest in EC cells. However, CDK 1 and 2 are functional in all four stages of the cell cycle, and dinaciclib has been reported to cause an accumulation of cells in the G1 phase in murine neuroblastoma models [49], S phase accumulation in metastatic melanoma cells [50], and the G2/M phase in triple negative breast cancer and ovarian cells [44,51] and mitotic arrest in follicular thyroid cancer [52]. High content image analysis in EC cell lines revealed that dinaciclib caused an accumulation of cells in G2/M in these cells. Analysis of MPM2, a phospho-epitope present in numerous proteins during mitosis, in these cells did not indicate an increase of cells in the M phase, suggesting that the dinaciclib-induced cell cycle blockade prevented entry into mitosis.

MPM2 analysis revealed the loss of a large 250 kDa phospho-protein, previously described as Pol II pSer2 [41], in treated cells, which was confirmed as Pol II pSer2 using a highly specific antibody. CDK9, a component of the P-TEFb transcription elongation complex [53], is a known target of dinaciclib, and its inhibition by the CDK inhibitor blocked Pol II pSer2 phosphorylation and thus transcription. Importantly, the concentration of dinaciclib required to inhibit Pol II pSer2 phosphorylation was identical to that required to significantly reduce cell viability. The bimodal effect of dinaciclib can therefore be achieved simultaneously.

Inhibition of CDK9 and thus transcription can result in a concomitant reduction of oncogene and/or anti-apoptotic gene expression. The anti-apoptotic regulator Bcl-2 is a known proto-oncogene in EC, while decreased expression of Mcl-1, an anti-apoptotic Bcl-2 gene family member, has been associated reduced EC cell viability following sorafenib treatment [37]. Here, dinaciclib caused a significant reduction in Bcl-2 expression, which, through subsequent loss of inhibition of the pro-apoptotic proteins Bax and Bak, is likely to account for Ishikawa cell death. Surprisingly, however, despite a reduction in cell proliferation, significant decreases in the expression of both Bcl-2 and Mcl-1 following dinaciclib treatment did not coincide with apoptosis in HEC-1A cells, possibly due to a compensatory effect of other anti-apoptotic proteins such as Bcl-Xl [54], or suggesting that other processes inhibited by dinaciclib were responsible for its effects.

The evaluation of dinaciclib with cisplatin in combination treatments demonstrated only a very small positive effect over dinaciclib treatment alone. No antagonistic effects were observed, suggesting that dinaciclib could be considered in patients whose cancer is becoming refractory to cisplatin without abrogating the effects of ongoing platinum-based treatments.

The arguments for targeting CDKs including those targeted by dinaciclib (CDKs 1, 2, 5, 9, and 12) are well-established and compelling [9,55,56]. Pan-CDKis inhibit multiple CDKs and thus target multiple cellular processes simultaneously, suggesting that these agents could be broadly effective over a heterogenous genetic landscape, as was seen here, with consistent efficacy of dinaciclib across EC primary cells and cell lines. Early trials with pan-CDKis including flavopiridol and dinaciclib failed due to off-target effects limiting their therapeutic window and therefore efficacy [18]. However, toxicity issues with dinaciclib have been addressed with alternate dosing schedules, and the drug is currently under trial in combination with veliparib for patients with advanced solid tumors [20]; flavopiridol is also undergoing clinical reexamination in the form of an oral pro-drug [19]. Efficacy and dose-limiting toxicity are two sides of the therapeutic coin, and continued research in identifying the tumor types most sensitive to CDKis, while establishing methods of reducing their toxicity, will be critical in exploiting the potential of these powerful drugs in the clinic.

## 5. Conclusions

Together, our findings demonstrate the effectiveness of dinaciclib in decreasing the viability of primary tumor cells isolated from both Type I and II EC patients, which was recapitulated in cell models. The bimodal mechanism of dinaciclib action, namely blocking cell cycle progression and inhibiting Pol II phosphorylation, with a concomitant repression of the anti-apoptotic genes Bcl-2 and Mcl-1, accounted for this high level of efficacy. Whilst dinaciclib remains to be established in the clinic, in part due to systemic toxicity observed at effective dose levels, its low-nM potency lends it to being an effective payload for encapsulation into synthetic (micelles, liposomes) or semi-synthetic (engineered exosomes/exosome mimics) nanoparticles, which would enable its exploitation in medical oncology.

## Data Availability

Data are contained within this article and the Supplementary Material.

## Supplementary Materials

The following are available online at https://www.mdpi.com/2072-6694/13/5/1135/s1. Table S1: Drug IC50 values (µM) in EC cell lines, Table S2: Biopsy histology and IHC data, Figure S1: Apoptosis in EC cell lines, Figure S2: MPM2 levels, Figure S3: Staurosporine in EC cell lines.
