# Supplementary material for: Dinaciclib, a Bimodal Agent Effective against Endometrial Cancer
Source: Cancers (Basel). 2021 Mar 6;13(5):1135. doi: 10.3390/cancers13051135 (PMC7962054; doi:10.3390/cancers13051135)
Supplement: Supplementary file 1 [file cancers-13-01135-s001.pdf]

Supplementary Material: Dinaciclib, a Bimodal Agent Effective Against Endometrial Cancer

David Howard, David James, Kate Murphy, Jezabel Garcia-Parra, Belen Pan-Castillo, Stuart Rex, Annemarie Moul, Eilir Jones, Marc Bilbao-Asensio, Saul Michue-Seijas, Kerryn Lutchman-Singh, Lavinia Margarit, Lewis W. Francis, Paul Rees, Deyarina Gonzalez and R. Steven Conlan

Table S1. Drug IC50 values (μM) in EC cell lines.

Drug	Ishikawa	HEC-1A	HEC-1B	HEC-50
Dinaciclib	0.006 ± 0.001	0.009 ± 0.001	0.006 ± 0.001	0.009 ± 0.001
Flavopiridol	0.039 ± 0.006	0.084 ± 0.017	0.108 ± 0.016	0.101 ± 0.032
DRB	17 ± 1	42 ± 3	39 ± 3	41 ± 1
Cisplatin	11 ± 1	28 ± 3	24 ± 3	29 ± 1
Carboplatin	-	17	75 [43]	-
Doxorubicin	12	4	-	-
Paclitaxel	-	0.035	-	0.015

± means standard deviation.

Table S2. Biopsy histology and IHC data.

Biopsy	Stage	Grade	Histology	Subtype	ER	p53
B1	FIGO 1A	G1	endometrioid type adenocarcinoma	I	5/8 (3+2)	+++
B2	FIGO 1A	G1	endometroid type adenocarcinoma	I	6/8 (3+3)	++
B3	FIGO 1A	high	serous carcinoma	II	5/8 (2+3)	+++
B4	FIGO 1B LVSI present	G3	endometrioid type adenocarcinoma	II	8/8 (5+3)	+++
B5	FIGO 1B	G2	endometrioid type adenocarcinoma	I	8/8 (5+3)	+++
B6	FIGO 1A	G3	endometrioid type adenocarcinoma	II	8/8 (5+3)	+++
B7	FIGO 1B	G1	endometrioid type adenocarcinoma	I	8/8 (5+3)	+++
B8	FIGO 1A LVSI present	G1	endometrioid type adenocarcinoma	I	8/8 (5+3)	+++

ER = estrogen receptor, LVSI = Lymphovascular space invasion.

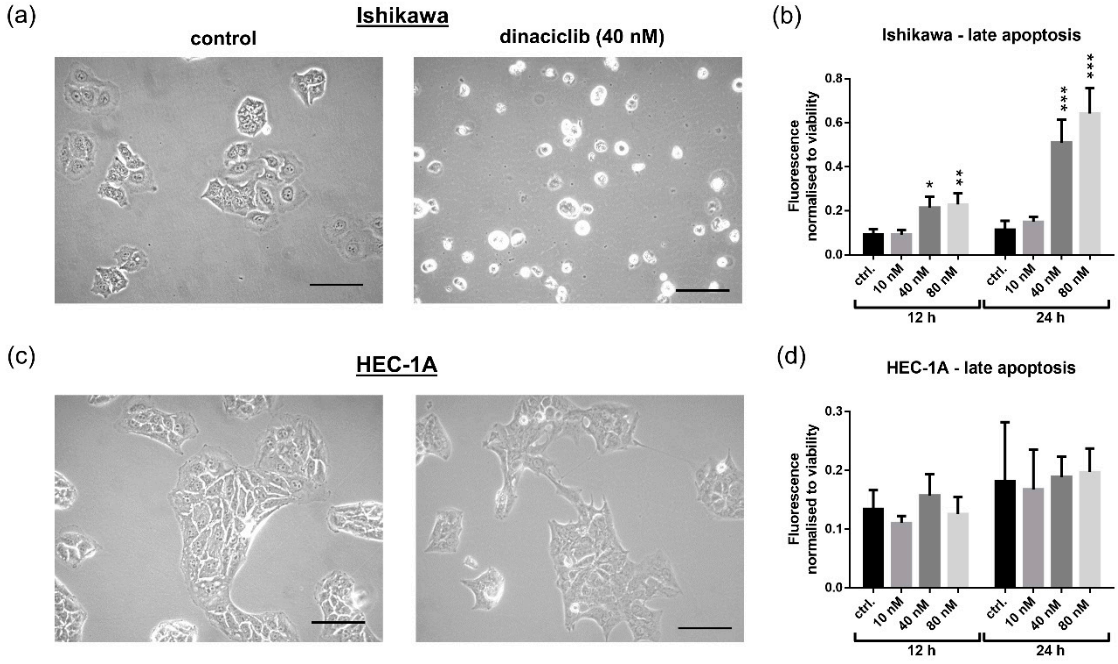

Figure S1. Dinaciclib induces apoptosis in Ishikawa, but not in HEC-1A cells. Phase contrast microscopy images of (**a**) Ishikawa and (**c**) HEC-1A cells treated with dinaciclib (40 nM) for 24 h. Cell morphologies indicate apoptosis in Ishikawa, but not HEC-1A cells. Scale bars = 50 µm. Late-stage apoptosis following 12 and 24 h treatments with dinaciclib at various doses in (**b**) Ishikawa and (**d**) HEC-1A cells. Late apoptosis levels were measured using the RealTime-Glo™ Annexin V Apoptosis and Necrosis Assay. Statistical significance was calculated using the ANOVA. * p value < 0.05 , ** p value < 0.01, *** p value < 0.001.

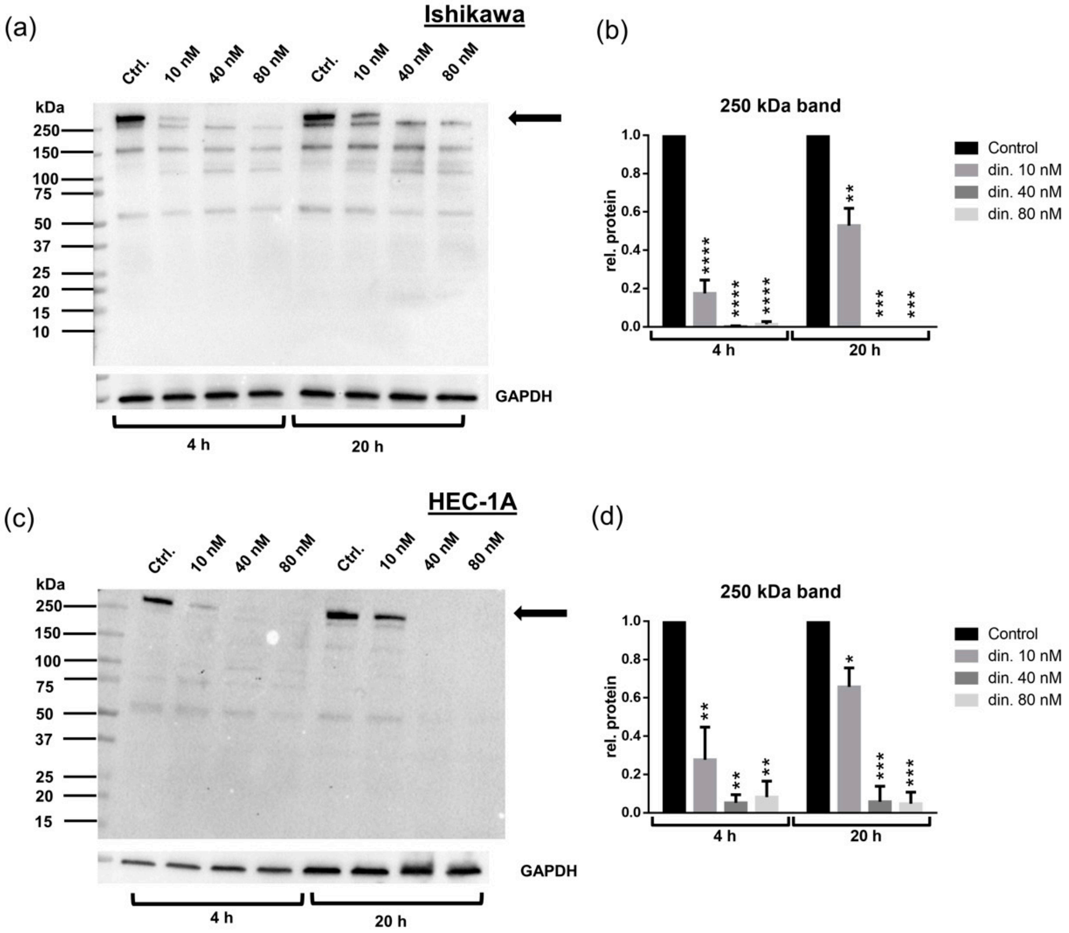

Figure S2. Dinaciclib reduces MPM2 levels. Ishikawa and HEC-1A cells were treated with dinaciclib at various doses (10, 40, or 80 nM) for 4 and 20 h and proteins probed with anti-MPM2. Representative blots for (**a**) Ishikawa, (**b**) HEC-1A with a 250 kDa protein band quantified via densitometry and normalized to GAPDH in (**c**) Ishikawa and (**d**) HEC-1A samples using three experimental replicates. * p value < 0.05 , ** p value < 0.01, *** p value < 0.001, **** p value < 0.0001.

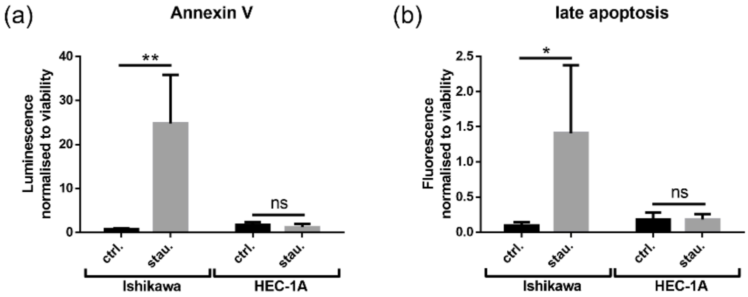

Figure S3. Apoptosis in Ishikawa and HEC-1A cells following treatment with 2 µM staurosporine (stau.) for 24 h. (**a**) Levels of relative Annexin V (indicative of apoptosis through binding of phosphatidylserine) in treated and control samples (**b**) Levels of relative late apoptosis in the same samples. Annexin V bound to cells and levels of late apoptosis were measured using the RealTime-Glo™ Annexin V Apoptosis and Necrosis Assay and normalized to sample viability. Statistical significance was calculated using the unpaired *t*-test. * p value < 0.05 , ** p value < 0.01,

Immunoblots used in Figures

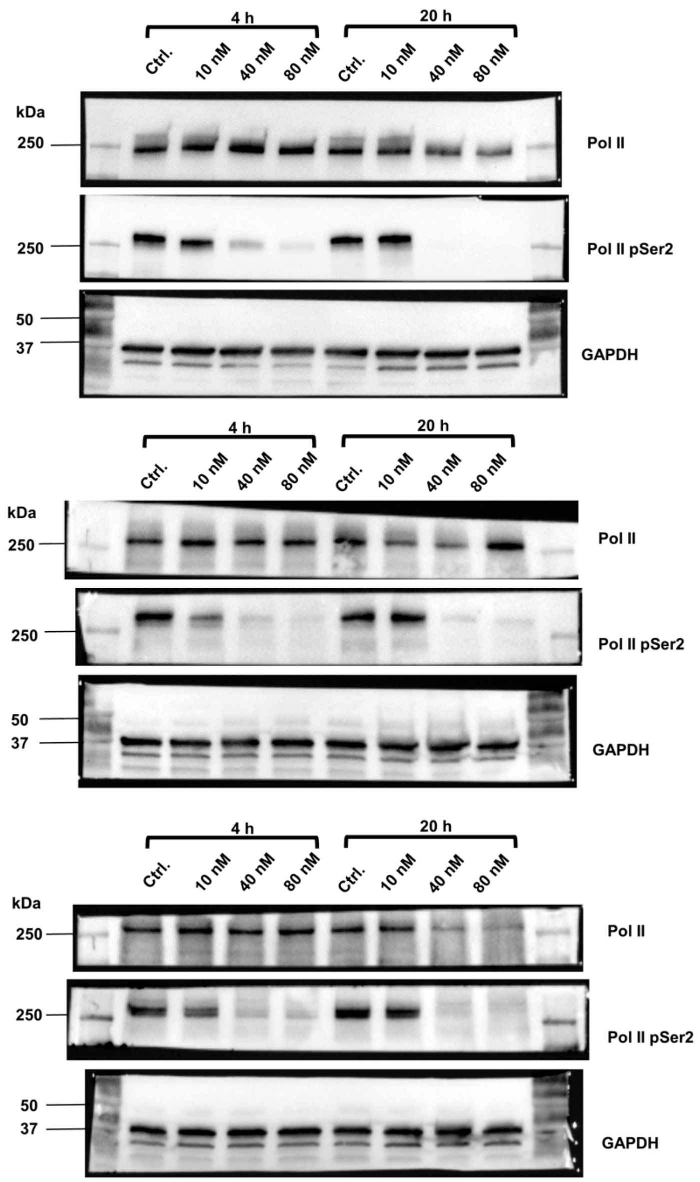

Figure A1. Immunoblots corresponding to data in Figure 5a and 5c.

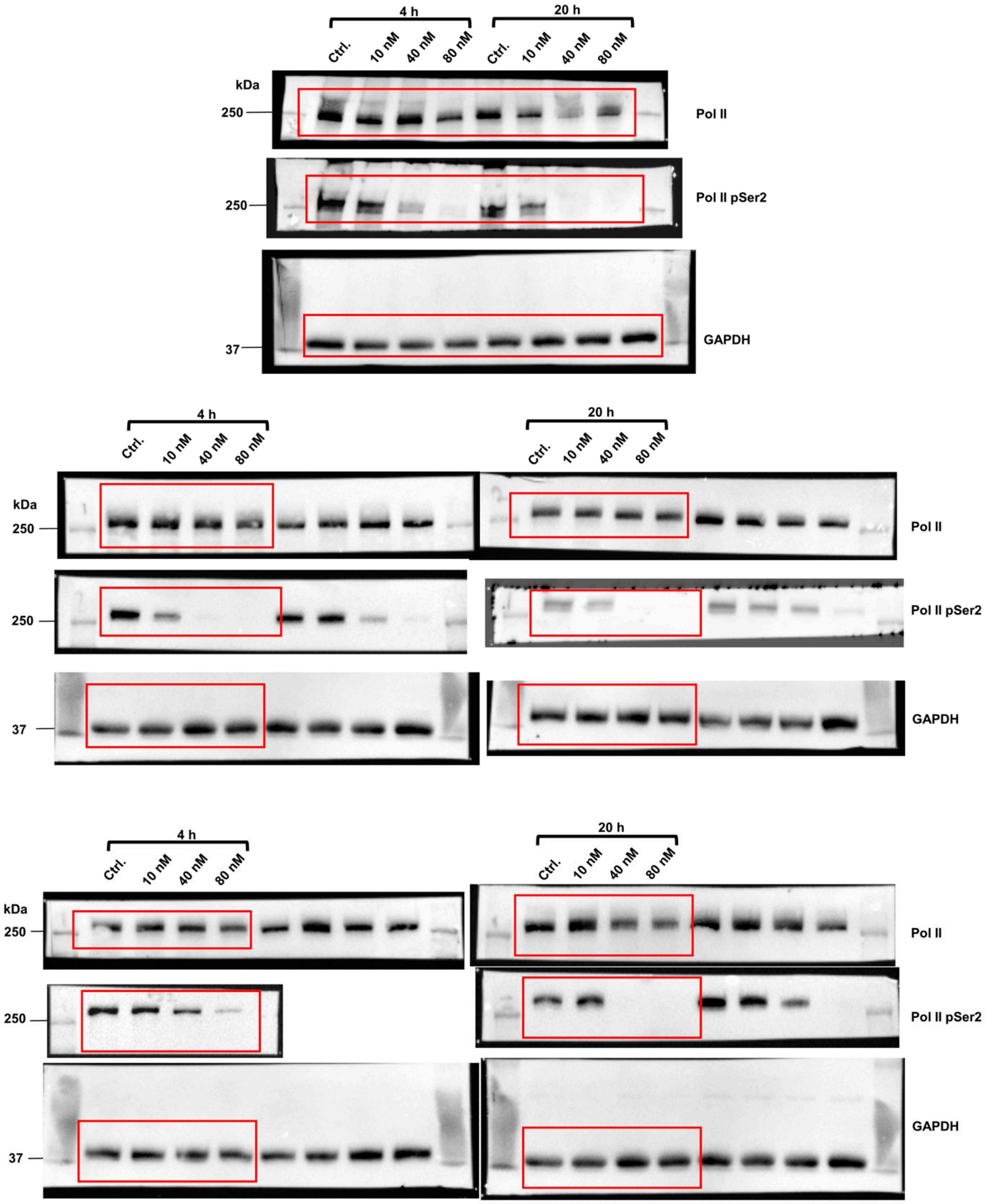

Figure A2. Immunoblots corresponding to data in Figure 5b and 5d. Red boxes indicate bands that were analyzed for these subfigures.

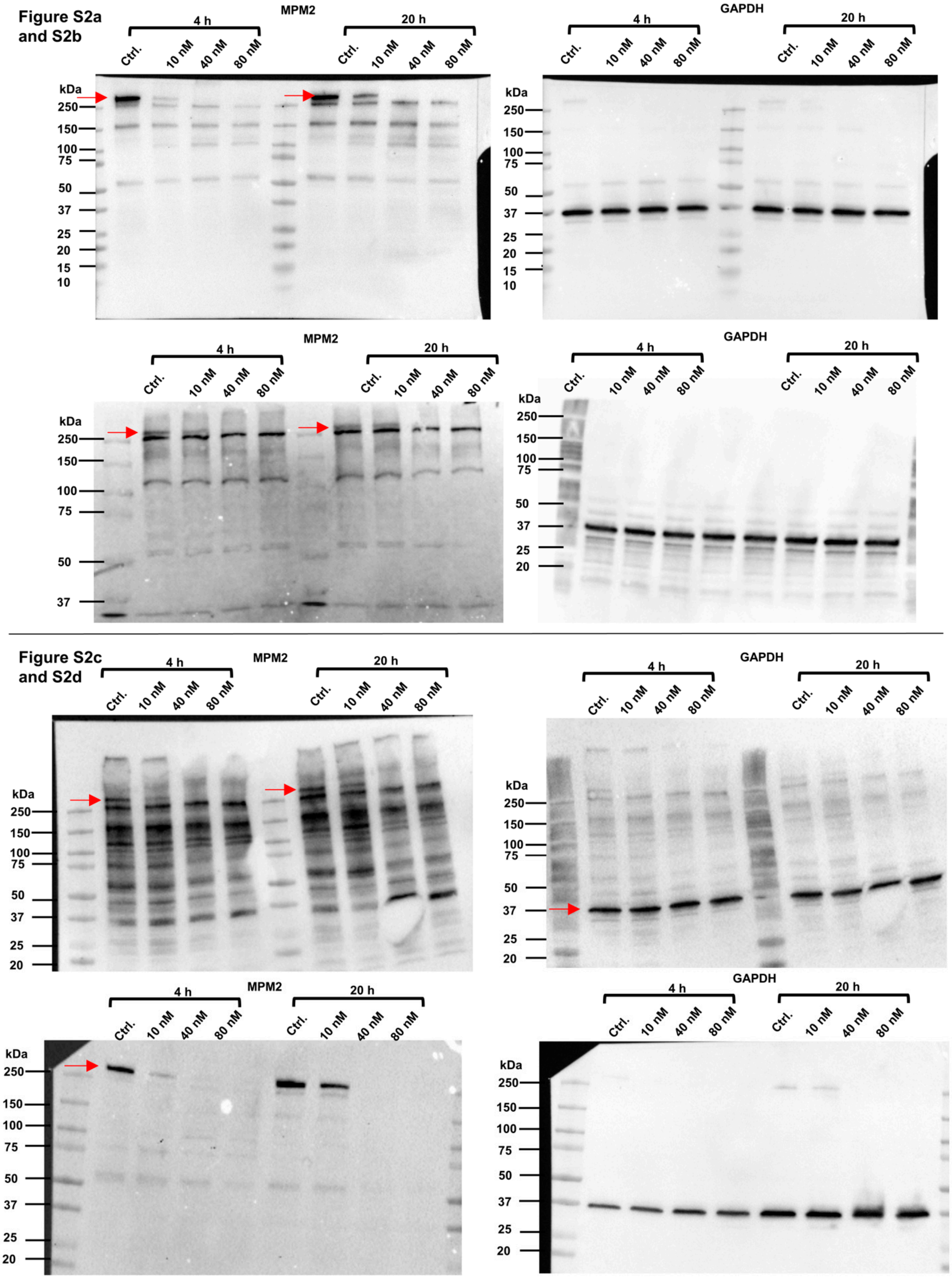

Figure A3. Immunoblots corresponding to data in Figure S2. Where multiple bands are present, red arrows indicate bands that were analyzed
